# Pilot Study of Evaluating Attitudes toward Childhood Immunization among Healthcare Workers in Japan

**DOI:** 10.3390/vaccines10071055

**Published:** 2022-06-30

**Authors:** Aya Saitoh, Yugo Shobugawa, Isamu Sato, Yuki Yonekura, Ai Kawabata, Akihiko Saitoh, Reiko Saito

**Affiliations:** 1 Department of Fundamental Nursing, Graduate School of Health Sciences, Niigata University, Niigata 951-8518, Japan; 2 Department of Active Ageing, Graduate School of Medical and Dental Sciences, Niigata University, Niigata 951-8510, Japan; yugo@med.niigata-u.ac.jp; 3 Yoiko-no Shounika Sato, Niigata 950-0983, Japan; yoiko136@gmail.com; 4 Department of Nursing Informatics, Graduate School of Nursing Science, St. Luke’s International University, Tokyo 104-0044, Japan; yyonekura@slcn.ac.jp; 5 Graduate School of Nursing, Yamanashi University, Yamanashi 400-8510, Japan; aikaw@yamanashi.ac.jp; 6 Department of Pediatrics, Graduate School of Medical and Dental Sciences, Niigata University, Niigata 951-8510, Japan; asaitoh@med.niigata-u.ac.jp; 7 Division of International Health, Graduate School of Medical and Dental Sciences, Niigata University, Niigata 951-8510, Japan; jasmine@med.niigata-u.ac.jp

**Keywords:** immunization, education, child, healthcare workers, vaccine hesitancy

## Abstract

Providing appropriate immunization information during the perinatal period is important for improving immunization rates among infants and children; however, the distribution of immunization information by healthcare workers (HCWs) is not standardized in Japan. We investigated HCWs’ attitudes toward childhood immunization and factors related to vaccine hesitancy. We conducted a cross-sectional descriptive survey of HCWs involved in childhood immunization in Niigata City, Japan, from November 2017 to January 2018. We assessed contextual, individual and group, and vaccine/vaccination-specific influences. Of 290 HCWs, 139 (47.9%) returned completed questionnaires. Most HCWs (87/139, 64.9%) reported providing immunization information verbally to parents; 51/87 (58.6%) spent fewer than five minutes doing so. Pediatricians provided vaccines based on the parents’ best interest, whereas public health nurses and midwives emphasized government policy. Nurses had greater hesitancy related to personal perceptions and social/peer factors than pediatricians (*p* < 0.001). Nurses were significantly more likely than pediatricians to suggest that children receive more shots than necessary (*p* < 0.01). Nurses tended to have more negative attitudes toward vaccination and little awareness of immunization promotion compared to pediatricians. Thus, all HCWs involved in childhood immunization should receive sufficient information to provide timely and appropriate immunization to infants and children.

## 1. Introduction

During the 2019 coronavirus– (COVID-19) pandemic, the importance of immunization was well recognized. Immunization has been an essential strategy to decrease the spread of the virus globally and improve mortality and morbidity associated with COVID-19. However, some people have negative perceptions of immunization due to safety concerns and anxiety about adverse reactions to the new COVID-19 vaccines, which were created in a short period of time. Vaccine hesitancy or refusal has become one of the most important and controversial issues in recent years [1]. Vaccine hesitancy is defined based on the Strategic Advisory Group of Experts on Immunization (SAGE) as a “delay in acceptance or refusal of vaccination despite the availability of vaccination services” [2]. It is a complex and context-specific phenomenon, varying by time, place, and vaccine, and it is influenced by factors such as complacency, convenience, and confidence [3].

Healthcare workers (HCWs) are expected to be aware of the risks and benefits of vaccination as well as the risks of vaccine-preventable diseases (VPD), and they should be able to communicate that information to caregivers in the most appropriate manner. Many developed countries have their own communication tools and provide parents with standardized education related to immunization. For example, education on infant immunization is systematically and routinely provided in the United States [4]. In other developed countries, informing parents of the risks and benefits of vaccines is the usual approach and is legally mandated [1,5,6]. However, in Japan, there are various ways to convey such information, and the content of immunization education varies among HCWs [7]. This is most likely because individual HCWs did not receive sufficient education related to immunization when they were trained.

Our previous study in Japan from 2014 demonstrated the deficiencies in the available infant immunization content in terms of both quality and quantity [8]; specifically, parents were unable to make optimal decisions on whether to administer voluntary vaccines to their children after receiving such information [8]. Although studies have identified HCWs as important sources of immunization information [9,10], understanding their attitudes toward vaccination is crucial. Researchers have reported that HCWs lack the confidence to provide vaccination information and recommendations to their patients [11,12,13,14]. A study demonstrated that HCWs in some European countries, including Croatia, France, Greece, and Romania, displayed vaccination hesitancy [15]. There also exists a strong relationship between the knowledge and attitudes of HCWs regarding vaccines and their vaccine recommendations for their patients [16]. If HCWs are hesitant, they might recommend vaccines less frequently or undermine patients’ confidence in them, thus contributing to patients’ vaccine hesitancy [11]. On the other hand, increased knowledge and positive attitudes toward vaccination among nurses have been positively associated with increased vaccination coverage [17]. Additionally, a positive association between nurses’ own vaccination status and their reported promotion of vaccinations to their patients has been reported [14,17].

The perceptions and attitudes of HCWs toward immunization can vary among professionals [18]. Although there have been studies on the differences in attitudes among providers toward immunization [9,13], few studies have characterized the nature and extent of vaccine hesitancy among HCWs. Therefore, this study investigated current practices in childhood immunization education in Japan and clarified HCWs’ attitudes toward childhood vaccination, focusing on vaccine hesitancy.

## 2. Materials and Methods

### 2.1. Study Design and Setting

This was a cross-sectional, descriptive survey of HCWs in Niigata City, Japan, which has a population of approximately 800,000. In Japan, most vaccines are required under the national immunization law, whereas a few, including the mumps and influenza vaccines, have been categorized as voluntary vaccines that are not mandated by the Japanese immunization law, and these require out-of-pocket payments. The vaccination rates in Niigata City are equivalent to the Japanese average rates [18]. HCWs involved in children’s vaccination were recruited for this study, including pediatricians, midwives, public health nurses, and registered nurses working at the hospital. The study period was 3 months, from 1 November 2017 to 31 January 2018. Although immunization information is mainly provided by pediatricians in Japan, the earliest information is provided by midwives after birth. Respondents were recruited if they agreed to participate in the study. The self-administered paper-and-pencil questionnaire and a letter explaining the study were mailed to HCWs and their institutions. Responding to the questionnaire implied consent on the part of the respondent to participate in the study.

### 2.2. Survey Questions

We used a measure developed by the SAGE Immunization Working Group on Vaccine Hesitancy [16]. The questionnaire used in the study was adapted to the Japanese immunization system and socio-cultural background. The Working Group developed a matrix displaying the factors influencing the behavioral decision to accept, delay, or reject some or all vaccines under three categories: contextual, individual and group, and vaccine/vaccination-specific influences [6]. These categories helped frame survey questions to identify and address vaccination hesitancy. The current study used the 27 items in the vaccine hesitancy scale related to HCWs [16]. The survey took approximately 10 min to complete, and the items were rated on a five-point Likert scale, ranging from 1 (“strongly disagree”) to 5 (“strongly agree”). Participants’ demographics (age, employment status, education, number of children, and family structure), implementation of current immunization education for parents, and method of obtaining the latest immunization-related information were also recorded.

The survey questionnaire was developed by the study team and pilot-tested among immunization specialists, nurses, and physicians caring for perinatal women, newborns, and infants. The questions were subsequently revised to improve clarity.

### 2.3. Ethical Considerations

This study was approved by the Institutional Review Board of the Niigata university (Approval number: 2017-0023).

### 2.4. Data Analysis

Statistical analyses were performed using SPSS version 19.0 (Chicago, IL, USA). The distribution of background characteristics and current immunization education status among survey respondents was assessed using descriptive statistics. ANOVA tests were used to compare the scores among the HCWs. The Games–Howell test was used as a multiple comparison post hoc test. All tests were two-tailed, and the significance level was set at *p* < 0.05. 

Based on effect size of 0.25 (F-test), assuming a power of detection of 0.8 (β = 0.2), α = 0.1, the estimated sample size required for this study was 144.

## 3. Results

### 3.1. Study Subjects

Questionnaires were distributed to 290 HCWs, of whom 139 (47.9%) returned completed questionnaires: 43 responses were from pediatricians (30.9%), 35 from public health nurses (25.2%), 41 from midwives (29.5%), and 20 from registered nurses (14.4%; Table 1). The pediatricians (55.4 ± 12.1 years) were older than the nurses; the youngest were registered nurses (37.1 ± 10.4), followed by the public health nurses (41.6 ± 8.6) and midwives (47.8 ± 11.7).

### 3.2. Frequency and Time of Immunization Education by HCWs

First, the participants were asked whether they discussed childhood immunization interactively with caregivers. If so, they were asked in detail about their opinion on whether they provided sufficient verbal immunization information to caregivers, the time required to do so, and the handouts they used for the discussion. The results are shown in Table 2. In total, 87 (64.9%) HCWs reported providing verbal immunization information to parents. The time spent on the discussion was short; 58.6% spent less than 5 min, 39.5% spent 5 to 10 min, and 16.1% spent more than 10 min. Most (83.9%) used immunization handouts including booklets for mothers, handouts from local governments, and information from pharmaceutical companies.

### 3.3. Immunization Information Sources among HCWs

We evaluated how HCWs obtained immunization information (Table 3). The internet was the main source of information for 51.2% of pediatricians and 63.9% of public health nurses. The pediatricians reported primarily using academic websites, such as the Japan Pediatric Society (JPS; 19.5%), Know VPD Protect Our Children (non-profit organization; 12.2%), and commercial websites for physicians (9.8%). In contrast, public health nurses often referred to public websites, including those of the Ministry of Health, Labor, and Welfare (27.8%), the JPS (11.1%), and the National Institute of Infectious Diseases (5.6%). The registered nurses reported using mass media, including television news and newspapers, as the top source (35.7%) of their information, followed by the internet (28.6%). In contrast, the midwives listed other HCWs (52.5%), followed by immunization seminars (47.5%), as the source of their information. The registered nurses used fewer information sources on average (1.0 ± 1.1) than the pediatricians (2.4 ± 3.4), public health nurses (2.1 ± 1.9), and midwives (1.9 ± 2.4).

### 3.4. Vaccination Hesitancy Category: Contextual

We compared average scores among the four HCW groups with respect to the influence of socio-cultural, institutional, and political factors (Table 4). The pediatricians (3.1 ± 1.0) were significantly more concerned about whether the decision by the government as to which vaccines to provide was in the citizens’ best interests, compared to public health nurses (3.9 ± 0.7, *p* < 0.001), midwives (3.9 ± 0.8, *p* = 0.002), and registered nurses (3.9 ± 0.7, *p* < 0.015). A lower perception of religion-based health risks of vaccination was reported in midwives (4.1 ± 0.7) versus pediatricians (4.7 ± 0.6, *p* < 0.003).

### 3.5. Vaccination Hesitancy Category: Individual and Group

Concerning personal views and social/peer environmental factors, significant differences were found between the perceptions of pediatricians, midwives, and public and registered nurses (Table 5). Regarding personal perceptions, pediatricians had lower hesitancy than midwives and public and registered nurses for three items: “Do you think vaccines overload the immune system?” (pediatricians vs. midwives, *p* = 0.001), “It is better for my child to develop immunity by getting sick than to get a shot” (pediatricians vs. midwives, *p* = 0.001; pediatricians vs. public health nurses, *p* = 0.014), and “Do you believe that it is better for the child to start receiving vaccines only when they are over one year of age?” (pediatricians vs. midwives, *p* = 0.013; pediatricians vs. registered nurses, *p* = 0.009). However, for the items regarding knowledge and information, such as “Do you feel you know which vaccines you should get for your children?” (pediatricians vs. midwives, *p* = 0.001) and “Do you feel you receive enough information about vaccines and their safety?” (pediatricians vs. midwives, *p* = 0.025), the midwives scored lower than the pediatricians (*p* < 0.01). For “Do you consider some vaccines to be more important than others?”, midwives scored lower than the pediatricians and public health nurses (pediatricians vs. midwives, *p* = 0.004; public health nurses vs. midwives, *p* = 0.003). Public health and registered nurses were significantly less aware of the importance of herd immunity, as indicated by their answers to questions such as “Do you believe that vaccines are still needed when diseases are rare?” (pediatricians vs. midwives, *p* = 0.028; pediatricians vs. public health nurses, *p* = 0.029). Nurses, including midwives, public health nurses, and registered nurses had a significantly lower perception of herd immunity by vaccination items, “I agree that it is important for everyone to get the recommended vaccines for themselves and their children” compared with pediatricians (pediatricians vs. midwives, *p* = 0.011; pediatricians vs. public health nurses, *p* = 0.001; pediatricians vs. nurses, *p* = 0.028).

### 3.6. Vaccination Hesitancy Category: Vaccine-Specific Issues

In terms of factors related to vaccine-specific issues, compared to pediatricians, public health nurses, midwives, and registered nurses were significantly more concerned that children receive more shots than are good for them (pediatricians vs. midwives, *p* = 0.007; pediatricians vs. public health nurses, *p* = 0.001, pediatricians vs. nurses, *p* = 0.003), as shown in Table 6.

## 4. Discussions

The present study examined current vaccination practices and sought to clarify attitudes that engender vaccination hesitancy across different HCWs in Japan. These findings are important for identifying vaccine-related concerns, understanding the attitudes among HCWs, and finding which HCWs are the most hesitant toward childhood vaccinations, which illustrates different reasons for vaccine hesitancy among HCWs.

Regarding childhood immunization, the information sources among HCWs vary, stemming from differing educational backgrounds on immunization. Although our results demonstrated that pediatricians have access to reliable information resources, nurses rely on different resources, including mass media and information from other HCWs, which might be biased. In general, nurses have fewer opportunities to learn about immunization during school or post-graduate training. The findings of this study indicate that the HCWs need to improve their knowledge through increased access to reliable immunization information. A previous study showed that among HCWs, greater intentions to vaccinate and more favorable attitudes toward vaccination were associated with higher awareness and positive beliefs that were more aligned with scientific evidence [19]. The results of this study concur with this prior study. Unfortunately, no official, standardized learning tool for immunization education for HCWs is currently available in Japan; hence, HCWs inevitably pursue individual learning opportunities using different resources, which could result in an increased information gap. Securing educational opportunities for HCWs through immunization policies should be considered as a way to create well-informed HCWs.

We demonstrated that more than half of HCWs provided 1 to 5 min of immunization education to parents or caregivers; however, this duration is considered insufficient [8]. In a nationwide survey in the United States, physicians and nurses reported spending an average of 3 min discussing vaccine risks and benefits with parents [20]. In the current study, midwives provided immunization education to parents for 6–10 min, which was more than the time spent by any other type of HCWs. Studies have reported that when HCWs have accurate immunization information based on scientific evidence, along with a positive attitude toward vaccination, they are more likely to engage in and provide positive information regarding vaccination [20,21,22,23]. Considering all the different sources of immunization information, updating the relevant information with the latest evidence-based knowledge is crucial. Nurses have more frequent opportunities to educate parents about immunization than other HCWs, such as during newborn home visits and one-month post-partum check-ups. As such, nurses have a significant impact on the vaccination decision-making of parents and caregivers, even for those who believe vaccines are unsafe [24,25]. Therefore, nurses should acquire accurate and reliable scientific information regarding an immunization to improve parents’ confidence in vaccination. This needs to be provided by other HCWs, especially physicians who interact with nurses.

All HCWs involved in infant immunization should receive sufficient information to serve as educators and guarantee that the instructions provided to parents are of high quality. The duration, content, location, and individuals responsible for immunization education varied across HCWs, indicating that immunization information is neither standardized nor efficiently communicated in Japan. A study suggested that a team approach to immunization education by physicians and nursing staff needs to be recognized, coordinated, and strengthened [21]. Thus, multidisciplinary collaboration is required to achieve a unified education system. The HCWs involved in immunization should receive standardized instructions to ensure that they provide consistent guidance. Therefore, the content and implementation of educational programs should be improved, and information should be shared among the concerned disciplines.

In the current study, for the first category, contextual, pediatricians were found to be more concerned about the national vaccine policy than all nursing professionals including midwives, public health nurses, and registered nurses. One possible reason for this is that nursing professionals might be less interested in and knowledgeable about national vaccine policies. Interestingly, midwives were significantly less likely to recognize the health risks stemming from non-vaccination for religious and cultural reasons. Several studies have shown that midwives have specific perceptions and attitudes toward immunization compared to the relatively more institutionalized sentiments of other HCWs. For example, whereas pediatricians were found to be supportive of immunization programs, midwives reported various levels of support [19,21,26,27], and they were often more concerned about trust in vaccines and vaccine methods [28].

Regarding the second subcategory, namely personal cognition and socio-environmental factors, there were significant differences between public health nurses, midwives, registered nurses, and pediatricians in items related to knowledge, such as concern about immune overload, lack of awareness about the severity of natural infection, and poor understanding of vaccination schedules. Nursing professionals had more concerns than did pediatricians. It was further found that pediatricians had lower vaccination hesitancy than did nursing professionals for most items.

This current finding is consistent with the results of previous studies regarding the concerns of midwives [22] and reports that public health nurses and midwives can have significantly different opinions from pediatricians on items related to knowledge, such as awareness of the severity of natural infections, social defense, and herd immunity. Previous studies have reported that knowledge acquisition and positive beliefs by HCWs may lead to positive changes among patients [17,23,29]. Therefore, educating HCWs is necessary to secure the quantity and quality of knowledge related to immunization. Furthermore, we found that both midwives and public health nurses tended to have similar concerns.

In terms of the third category, factors directly related to vaccination, the nurses’ concerns about over-vaccinating children were also significantly higher than the pediatricians’ concerns. A previous study in Canada showed that most midwives believed that vaccination begins too early in life [8]. Further verification is needed to determine whether this is due to differences in knowledge or related to other factors.

This study has some limitations. First, the number of subjects in the study was small, which may have decreased the likelihood of detecting potential differences, and the generalizability of the findings might be limited. A further study with a larger number of study subjects in different geographic areas needs to be conducted. Second, the study population was recruited from one geographical area, limiting the generalizability of the findings to all HCWs in Japan. Third, potential selection bias may exists given the limited response rates and sample size of the survey, which might have affected the validity of the results. The findings, therefore, may not represent the trends among HCWs in Japan. Fourth, the age of the HCWs differed significantly; differences in experience among professionals may affect the knowledge and attitudes of HCWs. Finally, causal relationships cannot be identified in this cross-sectional study; thus, further longitudinal research is necessary to determine professional attitudes that affect the actual patient vaccination rate and the extent to which these correlate with HCWs’ knowledge of vaccination. However, despite these limitations, this study illuminates the nature of hesitancy among HCWs involved in immunization in Japan. Directions for future study include linking provider attitudes, knowledge, and educational practices directly to outcome variables, such as vaccine acceptance, coverage, or parental satisfaction with immunization services.

In conclusion, most HCWs reported spending time communicating about vaccines; however, the time was limited, and they did not appear to have received sufficient information about immunization in infants and children. Overall, nurses, especially registered nurses, tended to have more negative attitudes toward vaccination and limited awareness of immunization promotion compared to pediatricians. All HCWs involved in the immunization of infants and children need to receive adequate information so that they can serve as educators, thus ensuring the superior quality of the instructions provided. Future research could explore specific factors related to vaccine hesitancy, providing insight into the differences in hesitancy and the need for a more comprehensive education for each type of HCW.

## Figures and Tables

**Table 1:** Characteristics of study participants.

	Pediatrician		Public Health Nurse		Midwife		Registered Nurse	
Total	43		35		41		20	
Age (±SD)	55.4	(12)	41.6	(8.6)	47.8	(11.7)	37.1	(0.3)
Education level (%)								
Vocational school	0		12		9		4	
Junior college	0		7		16		3	
College graduate/graduate school	43		16		16		13	
Affiliation (%)								
Private hospital	7		0		2		5	
University hospital	13		1		11		14	
Public hospital	4		0		0		0	
Clinic	19		0		9		0	
Home visiting	0		0		10		0	
Public health center	0		34		5		1	
Maternity home	0		0		4		0	

**Table 2:** Immunization education provided by each healthcare worker.

	Total		Pediatricians		Public Health Nurses		Midwives		Registered Nurses		*p* ^2^
	(*n* = 139)		(*n* = 43)		(*n* = 35)		(*n* = 41)		(*n* = 20)		
	*n* ^1^	%	*n*	%	*n*	%	*n*	%	*n*	%	
Discussion with HCWs about childhood immunization during prenatal period											
Yes	87	(64.9)	27	(62.8)	27	(77.1)	28	(68.3)	5	(25.0)	0.03
No	46	(35.1)	15	(34.9)	8	(22.9)	13	(31.7)	10	(50.0)	
Duration of immunization education preferred by parents											
<5 min	51	(58.6)	20	(46.5)	17	(48.6)	9	(22.0)	5	(25.0)	0.01
5–10 min	23	(39.5)	5	(11.6)	4	(11.4)	14	(34.1)	0	(0.0)	
>10 min	14	(16.1)	2	(4.7)	6	(17.1)	5	(12.2)	1	(5.0)	
Providing written immunization materials by HCWs											
Yes	73	(83.9)	21	(48.8)	24	(68.6)	24	(58.5)	4	(20.0)	0.71
No	14	(16.1)	6	(14.0)	3	(8.6)	4	(9.8)	1	(5.0)	

^1^ Excludes no response. ^2^ Chi square test. HCWs: Health Care Workers.

**Table 3:** Source of immunization information ^1^.

	Total	Pediatricians		Public Health Nurses		Midwives		Registered Nurses	
	(*n* = 139)	(*n* = 43)		(*n* = 35)		(*n* = 41)		(*n* = 20)	
	*n*	*n*	(%)	*n*	(%)	*n*	(%)	*n*	(%)
Yes	125	37	(86.0)	34	(97.1)	38	(92.7)	16	(80.0)
Internet	61	21	(51.2)	23	(63.9)	13	(32.5)	4	(28.6)
Immunization education seminar	42	12	(29.3)	10	(27.8)	19	(47.5)	1	(7.1)
Other professionals	40	8	(19.5)	10	(27.8)	21	(52.5)	1	(7.1)
Media	37	7	(17.1)	14	(38.9)	11	(27.5)	5	(35.7)
E-learning	34	12	(29.3)	15	(41.7)	4	(10.0)	3	(21.4)
Specialized books	25	21	(51.2)	0	(0.0)	3	(7.5)	1	(7.1)
International journals	13	11	(26.8)	2	(5.6)	0	(0.0)	0	(0.0)
Commercial journals	6	2	(4.9)	0	(0.0)	3	(7.5)	1	(7.1)
Official journals of scientific societies	2	2	(4.9)	0	(0.0)	0	(0.0)	0	(0.0)
No	14	6	(14.6)	1	(2.8)	3	(7.5)	4	(28.6)
Total sources/person		2.37		2.14		1.87		1	

^1^ Multiple choice response.

**Table 4:** Contextual influences among healthcare workers.

		Scores *	Total		Pediatricians		Public Health Nurses		Midwives		Registered Nurses		
			(*n* = 139)		(*n* = 43)		(*n* = 35)		(*n* = 41)		(*n* = 20)		
		(Range)	Mean	(SD)	Mean	(SD)	Mean	(SD)	Mean	(SD)	Mean	(SD)	*p*
1	Do you trust that your government is making decisions in your best interest with respect to what vaccines are provided?	(1–5)	3.7	(0.9)	3.1	(1.0)	3.9	(0.7)	3.9	(0.8)	3.9	(0.7)	<0.001
2	Do you think patients are risking their health or the health of their child if they do not take a vaccine because of religious reasons?	(1–5)	4.4	(0.8)	4.7	(0.6)	4.4	(0.6)	4.1	(0.9)	4.1	(0.7)	0.003
3	Do you think governments are “pushed” by lobbyists or industry to recommend certain vaccines?	(1–5)	2.8	(0.9)	2.8	(0.9)	2.9	(0.7)	2.7	(0.9)	2.8	(1.1)	0.7
4	Do you trust pharmaceutical companies to provide safe and effective vaccines?	(1–5)	3.8	(0.8)	3.8	(0.8)	3.6	(0.9)	3.9	(0.8)	3.8	(0.8)	0.67

* Scores: 1 = Strongly Disagree, 2 = Disagree, 3 = Neither agree nor disagree, 4 = Agree, 5 = Strongly Agree.

**Table 5:** Scores of individual social group influences among healthcare workers.

		Scores *	Total		Pediatricians		Public Health Nurses		Midwives		Registered Nurses		
			(m = 139)		(*n* = 43)		(*n* = 35)		(*n* = 41)		(*n* = 20)		
		Range	Mean	(SD)	Mean	(SD)	Mean	(SD)	Mean	(SD)	Mean	(SD)	*p*
1	Do you think vaccines overload the immune system?	(1–5)	2	(0.7)	1.7	(0.5)	2	(0.6)	2.3	(0.7)	2.4	(1.0)	<0.001
2	It is better for my child to develop immunity by getting sick than to get a shot.	(1–5)	1.8	(0.9)	1.4	(0.6)	1.9	(0.9)	2	(0.9)	2.2	(1.0)	<0.001
3	Do you believe there are other (better) ways to prevent diseases apart from vaccination?	(1–5)	2.1	(0.9)	1.9	(1.0)	2.1	(0.7)	2	(0.9)	2.5	(0.9)	0.172
4	Do you believe that it is better for the child to start receiving vaccines only when they are over one year of age?	(1–5)	1.7	(0.7)	1.4	(0.5)	1.7	(0.7)	1.9	(0.8)	2.3	(0.8)	<0.001
5	Do you feel that you know which vaccines you should get for your children?	(1–5)	4.2	(1.0)	4.5	(0.8)	4.3	(0.7)	3.9	(1.1)	3.6	(1.2)	0.002
6	Do you feel you get enough information about vaccines and their safety?	(1–5)	3.6	(0.9)	4	(1.0)	3.6	(0.8)	3.4	(0.8)	3.2	(1.1)	0.025
7	Do you consider that some vaccines are more important than others?	(1–5)	3.5	(0.9)	3.8	(0.9)	3.8	(0.7)	3.1	(1.0)	3.6	(0.9)	0.003
8	I trust the information I receive about shots.	(1–5)	4	(0.5)	3.9	(0.6)	4.2	(0.4)	4	(0.4)	3.9	(0.6)	0.129
9	Do you believe that vaccines are still needed when diseases are rare?	(1–5)	4	(0.8)	4.3	(0.7)	3.9	(0.8)	4	(0.9)	3.6	(0.9)	0.006
10	I agree that it is important for everyone to get the recommended vaccines for themselves and their children.	(1–5)	4.2	(0.7)	4.6	(0.5)	4.1	(0.7)	4.2	(0.7)	3.9	(0.7)	<0.001
11	Do you think it’s important to get a vaccine to protect those that cannot get vaccinated?	(1–5)	4.2	(0.8)	4.6	(0.6)	3.8	(1.0)	4.2	(0.8)	4	(0.7)	<0.001

* Scores: 1 = Strongly Disagree, 2 = Disagree, 3 = Neither agree nor disagree, 4 = Agree, 5 = Strongly Agree.

**Table 6:** Vaccine-specific issues.

		Scores *	Total		Pediatricians		Public Health Nurses		Midwives		Registered Nurses		
			(*n* = 139)		(*n* = 43)		(*n* = 35)		(*n* = 41)		(*n* = 20)		
		(Range)	Mean	(SD)	Mean	(SD)	Mean	(SD)	Mean	(SD)	Mean	(SD)	*p*
1	Do you believe vaccines are safe for yourself? For your child/children? For those in your community?	(1–5)	4.0	(0.7)	4.2	(0.7)	4.0	(0.5)	3.8	(0.7)	4.2	(0.6)	0.074
2	New vaccine trials are not required to meet the same rigorous standards as any normally prescribed drug.	(1–5)	2.5	(0.9)	2.3	(0.9)	2.4	(0.7)	2.8	(1.0)	2.7	(0.7)	0.078
3	Do you fear the pain to you/your child or fear the needles when receiving a vaccine, and does this make you hesitate be to immunized?	(1–5)	2.4	(1.0)	2.4	(1.2)	2.4	(0.9)	2.3	(1.1)	2.1	(0.9)	0.827
4	Is the vaccination process welcoming?	(1–5)	3.4	(1.0)	3.1	(1.0)	3.7	(0.8)	3.4	(0.9)	3.3	(0.8)	0.086
5	Children get more shots than are good for them.	(1–5)	2.1	(0.9)	1.6	(0.7)	2.2	(0.6)	2.2	(1.0)	2.7	(0.9)	<0.001
6	It is better for children to get fewer vaccines at the same time.	(1–5)	3.0	(1.1)	2.9	(1.5)	3.1	(1.0)	2.8	(1.0)	3.1	(0.7)	0.717

MD: physician, MW: midwife, PHN: public health nurse, RN: registered nurse. * Scores: 1 = Strongly Disagree, 2 = Disagree, 3 = Neither agree nor disagree, 4 = Agree, 5 = Strongly Agree.

## Data Availability

Not applicable.
